# Staff perspectives on poor mental health in secondary school students: an increasing problem handled with insufficient resources

**DOI:** 10.3389/fpubh.2024.1292520

**Published:** 2024-03-01

**Authors:** Åsa Svensson, Maria Warne

**Affiliations:** Department of Health Sciences, Mid Sweden University, Östersund, Sweden

**Keywords:** adolescent, focus group, teacher, school health promotion, school health services

## Abstract

**Introduction:**

An increasing number of schools are recognizing the importance of addressing students' mental health based on the association with educational outcomes and long-term health. The school organization and the members of the school staff play important but, in several ways, challenging roles in this work. The purpose of this study was to explore views of staff from schools and school health services on mental ill health among students and their own role in detecting and managing it.

**Methods:**

A qualitative study was conducted in a sparsely populated municipality in northern Sweden. In total, 40 participants from three secondary schools and the school health services participated either in focus groups or individual interviews. Participants were teachers, assistants, school nurses, school counselors and psychologists. Data were analyzed using thematic analysis.

**Results:**

The analysis revealed the main theme *Student mental ill health: an increasing problem handled with insufficient resources* and two subthemes, i.e., *Uncertainty in interpreting students' signs of mental ill health and the need to clarify roles and establish a supportive organization*.

**Conclusions:**

It was concluded that school staff were uncertain regarding how to interpret signs of mental ill health among students and required better knowledge and more resources to help students with mental ill health. A clearer organization and consensus regarding support for students with mental ill health were also necessary in light of the division of responsibilities between school staff and the school health services.

## 1 Introduction

Mental health issues are common among young people worldwide, and schools have long been recognized as having a key role in addressing poor mental health in children and young people, as a result of the connection to educational outcomes (1) and longer-term health (2). Data drawn from the Global Burden of Disease study in 2019 show that the global prevalence of mental disorders is 14% among 10- to 19-year-olds (3). Examples of groups of mental disorders that are common in 10- to 19-year-olds include anxiety disorders, attention-deficit/hyperactivity disorders, and depressive disorders. Not all mental health issues qualify as mental disorders, but they still impact the lives of young people negatively. It is important to note that mental disorders or problems and wellbeing can exist independently in the same person, as shown by, for example, Patalay and Fitzsimons (4). It is desirable for all children and young people to exhibit both a high level of wellbeing and the absence of mental health conditions. In the present study, the term mental ill health is used broadly to describe the absence of good mental health, including mental disorders as well as problems that do not meet clinical criteria.

In the Scandinavian countries, self-reported psychosomatic symptoms (measured with an index of eight items of subjective health complaints, e.g., headache, stomach-ache, feeling low and irritability) have increased since the mid-1980s and are especially prevalent among girls (5). In a survey in 2021/22, 77% of girls and 46% of boys aged 15 in Sweden reported having at least two of these subjective health complaints (such as stomachaches or feeling low) at least once per week; that is, girls reported almost twice as many health complaints as boys (6). Counteracting mental ill health among adolescents is important since this phenomenon negatively affects young people's school performance and future health (2, 7). Psychosomatic symptoms and other internalized mental health problems have been shown to predict subsequent school absence and dropout (8), and both externalized and internalized problems have a negative relationship with school performance (7, 9). During adolescence, some mental disorders make their debut, which makes it important to pay attention to early symptoms (10). Therefore, to avoid negative consequences in adulthood, it is crucial to promote mental health in adolescence. The increased prevalence of mental illness among young people has thus become an important issue in line with the UN's global sustainability goal 2030. Target 3.4 focuses on reducing mortality from non-communicable diseases and promoting mental health and wellbeing.

Many guidelines and manuals have been developed to help schools promote mental health among children and young people. In recent decades, guidelines and manuals have been developed at the national, regional and international levels. Globally, the WHO has developed guidelines for Health Promoting Schools (HPS) as well as guidelines with a specific focus on mental health (11). Two main directions for promoting adolescents' mental health in the school setting can be discerned: schools that focus on individual health behavior and schools that take a more holistic approach, in which context the settings approach permeates the work (12). St. Leger et al. (1) argued that preliminary evidence indicates that health work in the school setting is transitioning from health promotion in the school setting to a health-promoting setting. That is, schools are taking control over this process instead of the health sector. The settings approach originates from the WHO and specifically from the Ottawa Charter for Health Promotion (13). The conceptual framework used by this approach involves (a) an ecological model of health promotion, (b) a system perspective and (c) a whole system approach to development and change (14).

In line with the central role of schools in mental health promotion, studies have investigated teachers' role in student mental health. At the turn of the millennium, St. Leger (15) noted that “teachers are fundamental to the success of a health promoting school” (p. 81). In a study by Beames et al. (16), teachers generally agreed that part of their professional role involves promoting and preserving students' mental health, for example, by establishing an inclusive school environment that educates students about mental health and captures students' concerns. However, teachers may be uncertain regarding how to deal with students with mental health problems, and they may fear the risk of exacerbating the problems (16, 17). A study conducted in Brazil showed that teachers had difficulties distinguishing between students with mental health problems and those who exhibited normal behavior for their age. It was especially difficult to identify students who exhibited only internalized problems (18). Shelemy et al. (19) showed that teachers wanted advice regarding ways of identifying mental illness among students and preventing a situation from deteriorating. The teachers who participated in that study wanted interventions and resources pertaining to mental health that could be adapted to individual contexts. The cited studies highlighted the need for support to enable teachers to improve their mental health literacy and confidence with the goal of promoting the health of their students.

In addition to teachers, the school organization plays an important role in student mental health, which is in line with the notion of the school as a health-promoting setting. For Swedish children, school is compulsory from preschool (6 years old) to school grades 1 to 9 (7 to 15 years old). Swedish school law (2010:800) stipulates that school health services (SHS) must primarily be preventive and health promoting (20). The role of SHS is to support students' development with regard to the goals of education. Educators and student health staff contribute to this goal by working in interprofessional teams. The SHS must include staff with medical, psychological, psychosocial and special educational expertise, but no requirements have been stipulated regarding how this work should be organized (20). The organization of SHS differs across European countries. Nearly all EU countries have adopted SHS, but they vary significantly in terms of governance, organization, content and comprehensiveness (21). The SHS in Sweden is school-based, while many other countries identify it as a part of the health system. In Sweden and many other countries, the health professionals associated with to SHS tend to work in multiprofessional teams (21). In terms of The Swedish Association of Local Authorities and Regions (SALAR), it has been reported that many municipalities feature both a centrally organized student health system and certain staff employed by the school principal (22). The report concluded that the biggest challenge to student health was the increased prevalence of mental health issues among students alongside long-term school absences.

Mental ill health among young people, especially girls, is increasing. School is an important environment for students' health and learning and offers the opportunity to ensure equal conditions with regard to students' mental health. The school staff's view of young people's mental health and their responsibility, role and opportunities are central aspects of the ability of the school to play this important role in promoting students' mental health and preventing and detecting mental illness. However, school staff's experiences of this have not been widely studied. Qualitative research enables an in-depth exploration of school staff's perspectives and a more comprehensive understanding of the complexity of mental health problems in young people, and the school staff's views on their own conditions, strategies, and responsibilities in dealing with these issues.

## 2 Aim

The purpose of this study was to explore views of staff from schools and school health services on mental ill health among students and their own role in detecting and managing it.

## 3 Methods

The study was a qualitative study conducted in a sparsely populated municipality in northern Sweden. Data were collected in two rounds both before and after the implementation of a mental health intervention (Youth Aware of Mental Health [YAM]) for students in school grades 8–9 (14–15 years old); this intervention was implemented between 2019 and 2022 in all three secondary schools in the municipality. The present study presents a secondary analysis of the data that were initially collected with the goal of evaluating the YAM intervention. Since the participants discussed the topics analyzed in the present study in a similar way before and after the intervention, it was decided to conduct a secondary analysis on the data as a whole. The school staff that participated in this study did not have a role in implementing the YAM intervention.

### 3.1 Context

The study was conducted in a sparsely populated municipality in northern Sweden where the tourism industry is an important source of income. Three schools participated, i.e., schools A, B and C. All schools are located in small communities and provide services to students from a large geographical area. The schools are responsible for 150–500 students each, with classes ranging from preschool to year 9 (age 6–15). The schools share a common school health service (SHS). A student health team is also present at each school; these teams consist of staff from both the SHS and the school (e.g., the principal, school nurse, school counselor, and special education teacher). The municipality decided to implement YAM in the schools since problems with mental ill health had been identified among the population.

### 3.2 Participants and procedure

The target group for the study consisted of members of the school staff who worked with students in the grades participating in YAM as well as staff employed at the SHS. Participants were teachers, assistants, school nurses, school counselors and psychologists. The SHS included centrally employed staff at one or more specific schools. The researchers provided an information letter containing informed consent forms to the project manager of YAM to distribute to prospective participants. The project manager was responsible for contacting each school via the principal and the head of SHS to establish a time for focus groups. The principal, in turn, put the question of participation to the staff. Through contact with the head of the SHS, student health staff were invited to a separate focus group.

During the first round of data collection, which took place between November 2019 and February 2020, the researchers visited three schools and the SHS. The second round of data collection was conducted between February and March 2022, and in this round, the researchers used a video communication platform to collect data from the three schools and the SHS. The reason for adopting this approach was that in March 2020, restrictions for physical meetings were introduced as a result of the COVID pandemic, which in Sweden, as in many countries, came to affect society in a variety of different ways.

Data were primarily collected from focus groups, including one group each for the separate schools and the SHS in each round of data collection. In addition, in the first round, four people chose to participate in individual interviews conducted by telephone or via a video communication platform. In total, 40 participants participated in the research (see Table 1). The two rounds of data collection did not include the same participants due to changes in staffing; however, several people participated in both rounds. The focus groups varied in size from 4 participants to as many as 7 participants, with the focus group conducted with the SHS featuring the most participants (Table 1). The focus groups that took place in the form of a physical meeting were conducted in a room provided by the school where the focus groups could be undisturbed. The focus groups and individual interviews lasted ~60 min each (ranging from 47 min to 86 min).

**Table 1:** Participants in the focus groups and individual interviews, who were drawn from three different schools and the school health services (SHS) before and after a mental health intervention.

**2019, before the intervention**	**2022, after the intervention**
School staff:	School staff:
School A 3 participants in the focus group + 1 individual interview	School A 5 participants in the focus group
School B 4 participants in the focus group	School B 3 participants in the focus group
School C 5 participants in the focus group + 2 individual interviews	School C 5 participants in the focus group
SHS staff:	SHS staff:
7 participants in the focus group + 1 individual interview	4 participants in the focus group
In total, 23 participants	In total, 17 participants

The focus groups and individual interviews were based on an interview guide that contained questions concerning the occurrence of mental ill health among students, signs of mental ill health and teachers' competence and strategies. The interview guide differed to some degree between the first and second rounds of data collection. During the first round of focus groups and interviews, focus was more on general questions that did not pertain to the YAM intervention, for example about the participants' views of trends and signs of mental ill health among the students. In the second round of data collection there was more focus on the perceptions of the intervention (not included in this article). Examples of the questions asked are as follows: *How common is mental ill health among students? How can you discover that a student is mentally unwell? What happens when you discover that a student seems to be mentally unwell? How do you view your competence with regard to helping a student who is mentally unwell?* The two researchers were each responsible for their own area of focus in the interview guide and asked questions about that topic. The other researcher observed and was responsible for taking notes and ensuring that all questions were discussed.

All focus groups and interviews were recorded and then submitted to a professional transcription service that transcribed the collected data verbatim.

### 3.3 Ethical considerations

All participants received written information about the study. Before each focus group or individual interview began, the researchers reiterated the aim of the study and ensured that the people present had chosen to participate voluntarily. The participants were also informed that their data would be kept confidential and used only for the stated purpose. Those who chose to stay for the focus group or interview, or connected to the video communication platform gave their informed consent to participate. In the first round of data collection, participants additionally provided written informed consent. The study was conducted in line with the principles of the Declaration of Helsinki and Swedish legislation. Ethical approval was not required since participants chose to participate in the research in the context of their professions.

### 3.4 Analysis

The transcribed focus groups and interviews were analyzed using an approach to thematic analysis inspired by Braun and Clarke (23). The secondary analysis conducted for this study was based on a previous analysis of the same material that focused on the perceived effects of the YAM intervention. The two researchers had good knowledge of the material based on the initial analysis. For the present study, the data material drawn from the two rounds of data collection was considered in its entirety. The authors reread the transcriptions individually and created new preliminary themes based on the existing codes. The preliminary themes were then compared and discussed until consensus was reached concerning the most prominent meaning content. A comparative analysis was conducted between the themes and the text, and a main theme was created through dialog. The analysis thus resulted in one overarching theme and two subthemes.

The focus groups, interviews and analysis were conducted in Swedish, following which the final results and quotations were translated to English.

### 3.5 Researcher characteristics and reflexivity

The authors who conducted the focus groups, interviews and data analysis both have extensive previous experience in conducting research studies in the school environment, including focus groups and individual interviews with school staff and students. ÅS is originally from the municipality in which the study was conducted. MW has previous experience with the evaluation of municipal school activities. A reflexive approach was employed by each researcher during the process of data coding and in the joint discussions about the data.

## 4 Results

The analysis resulted in the main theme *Student mental ill health: an increasing problem handled with insufficient resources* and two subthemes, i.e., *Uncertainty in interpreting students' signs of mental ill health* and *the need to clarify roles and establish a supportive organization*.

### 4.1 Student mental ill health: an increasing problem handled with insufficient resources

Although the school staff identified different signs of students' mental ill health, they expressed doubts and sometimes mistrust regarding whether these issues truly signified mental ill health among the students. The amount of mental ill health among the students was sometimes overwhelming for the school staff, who agreed with staff from the SHS that mental ill health had become much more common in recent years. The school staff employed certain strategies when they suspected mental ill health in students. These strategies often involved communication with other staff, the student him/herself, and the student's peers and guardians. Even if the school staff dealt with such problems, they often lacked sufficient knowledge and did not consider the task of addressing mental health problems among students to be a part of their profession. The school staff requested more support from the SHS. Staff from the SHS believed that the school staff were able to manage student mental health effectively in the context of everyday activities and claimed that their own role was mainly to support the school staff in this work. The results are presented in detail under the two subthemes below.

### 4.2 Uncertainty in interpreting students' signs of mental ill health

The school staff mentioned that mental ill health is a trend in society, and they observed different signs of this phenomenon among the students. They generally observed more girls with mental ill health than boys but believed that girls showed clearer signs of this status. However, they found it difficult to understand which signs to take seriously and which were expressions regarding the student's socialization.

The analysis showed that the staff generally agreed that mental ill health had increased in recent years and was now more common among the students. However, they expressed uncertainty concerning the extent to which mental ill health was actually increasing and how much of that apparent increase that was due to students' increased willingness and daring to discuss this issue. The staff expressed positive attitudes toward the facts that people nowadays can discuss mental ill health more freely and that they have words to describe this phenomenon.

Respondent: Yes. Because it [mental illness] hasn't been talked about at all before, I think.

Respondent: No, but it is quite clear that it is increasing. I sometimes feel 'But oh, what has happened, that they feel so bad!'

Respondent: But then I also think it's like this when you get a language for it. When it comes to the surface, you talk about feeling bad in a different way. They didn't do that before. […] I don't know when it started, but it didn't used to exist.

Respondent: No. Back then, you were mentally ill, and it was almost a little ugly.

Respondent: So, they were careful not to talk about it too much, I feel. (School B)

The staff were unsure whether the students truly knew the meaning of these words and concepts pertaining mental ill health. The school staff noted that the students occasionally use terms such as panic disorder, which the staff instead interpret as nervousness. They questioned whether the students' statements concerning mental illness signified a real problem or represented more of a trend. Doubts therefore arose concerning how these different signs should be interpreted.

The problem of using different definitions was discussed by members of school staff and the SHS. The SHS requested a common language for mental ill health, since different ways of discussing this phenomenon makes it difficult to determine whether a problem is evident. One participant from the SHS claimed that students, school staff and staff from the SHS employ different languages to describe and have different views regarding mental ill health. Accordingly, mental ill health could be both underestimated and overestimated by students and school staff. Panic disorder was listed as one example of an overestimated problem, while eating disorders were described as an issue that was underestimated.

Panic disorder was described by the school staff as one of the easier conditions to interpret. “*There have been a lot of people who have had panic disorder, and it's very visible. It really becomes clear that this person is not well.”* An explanation of this fact can be that some school staff had been trained to observe panic attacks. On the other hand, the school staff questioned whether so many students had panic disorder. “*It feels very strange when several girls ... was it five in 1 day? That five girls got panic disorder on the same day.”*

The results of the analysis further highlight an aspect of distrust of students' mental ill health: the use of signs of mental ill health to obtain attention or status. This behavior was interpreted as a way in which the student could create an individual identity or satisfy a group norm. The school staff noted that students use mental ill health to some degree as a “sticker” that they can try on and then remove to determine whether it suits them or not.

Respondent: But then, sometimes it feels like that is also a status marker. “How much have I cut myself in relation to you?”

Respondent: I have noticed that in some discussions. They feel bad, but sometimes I also wonder, what does it stand for, fully? “Look, I've cut myself too. And I have cut myself this much.” Because some are very open about showing others, “look how I look.” While others try to hide it. So, that is also something…

Respondent: And I think it has become very different because I experienced when you had students who cut themselves 15 years ago, they never showed that they had cut themselves. Now, I think it's short sleeves and it's really like, well, “here I have all my scars.” So, that has changed too. (School B)

Signs that the school staff were already used to and felt more confident to interpret were changes in the students' relation to their studies. If students began to mismanage their studies or fall behind, this situation caused the school staff to become suspicious or worried. Other signs that were mentioned included situations in which a student stopped eating, did not seem to receive sufficient sleep or did not take care of his or her hygiene.

Respondent: Something else that you actually see and feel, that is, health. Because I know a lot of cases at this school where it's like, okay, this person stinks of sweat, they have bags under their eyes; it doesn't look like the person is sleeping, washing or anything like that. And I usually connect that to the fact that this person is unwell. Whether it's right or wrong, I don't know, but it's a classic warning sign that you stop taking care of yourself. (School C)

School staff expressed the suspicion that some students overdramatize normal events and create drama to receive attention from their peers, teachers, and parents. One explanation for these behaviors was that they were the result of absent parents. Some parents were described as too busy with their careers and smartphones to sit down and talk to their children. In one school, parents were described as both absent and demanding success in the context of both schoolwork and sports, which created great pressure for the students. Another interpretation of the reasons for students' over dramatization was that they were overwhelmed by their feelings and did not know how to interpret them. This situation was viewed as normal behavior for the age group in question and not necessarily as a sign of mental ill health.

Expressing and discussing mental ill health was viewed as being enhanced by social media, especially among girls. A Swedish influencer was identified as an example of how one individual inspired the students both to discuss their mental health problems and to frame themselves as unique persons, i.e., as a brand. This situation made it difficult for the school staff to determine which signs truly indicate mental ill health and which merely represent a way for the student to create an individual identity based on mental ill health in line with a role model.

Respondent: … I felt that there were so many students who expressed themselves in exactly the same way about how they felt. So, then I was surprised because I felt that this vocabulary that they use (...) And then I checked that blogger, and it turned out that much of what they said were exactly NN's words, how she felt. Her mental condition was what they reflected because there were five, six of them who expressed exactly the same symptoms and everything. (School B)

Peer pressure was observed by the school staff to contribute to mental ill health among the students. In particular, girls were seen to reinforce mental ill health mutually by discussing it frequently.

Respondent: I feel a little more that those who suffer from mental illness need to be separated. Because they support... they drag each other down instead. They think they support each other, but they pull each other down more than they support and help each other up. That's how I feel.

Researcher: Okay. Yes, that's interesting.

Respondent: Mm. They end up in a vicious circle.

Respondent: “Yes, I feel so bad today. Ouch, ouch, ouch, ouch. Yes, I feel bad today too. Yes, let's go and feel bad together for a while.” Now, I'm not saying that the whole school does it, but there are certain individuals who end up... seeking each other out. And of course, that's the way it is. But they are not always good for each other, these ones who are not doing so well. (School A)

The school staff further noted that not only peer pressure but even a contagion effect can be observed among the students, identifying panic attacks and self-harm as examples of “contagions” in the context of mental ill health at one school. Their suspicions regarding such a contagion effect were strengthened by the fact that several students exhibited signs of the same types of mental ill health. The examples given included self-harm, anxiety and eating disorders. These problems seemed to appear in waves among the students, building up and then subsiding until a new issue appeared. One school staff member related an experience that had occurred 1 year previously:

Respondent: The last class that finished the ninth grade there, when I look at that photo, it's... well, there were a few, maybe two girls, who didn't have signs of mental ill health.

Researcher: Hmm.

Respondent: And then I mean ... what I mean by mental ill health is that they possibly had anxiety, they cut themselves, they starved themselves, they had low self-confidence, they went to the counselor and talked. It was like an epidemic for a while at the beginning with that class, that they cut themselves, several of them. (...) With the boys, it doesn't express itself in the same way with self-starvation and cutting and anxiety and so on. (School A)

Gender differences related to mental ill health were discussed at most schools. Mental ill health was generally expressed in an internalized form among girls but an externalized form among boys, and more girls than boys discussed mental ill health with friends and staff. As one member of the school staff noted, “*They [girls] are often more eager to talk about how they feel, and their mood, and what is difficult, and so on.”*

On the other hand, school staff noted that it was easier to become aware of girls' mental ill health than boys' because it is more visible in girls. Girls discuss this issue, exhibit overt panic attacks and gather around each other when some of them signal mental health problems. In contrast, boys are more inclined to argue, act out, and fight, behaviors which were not always interpreted as signs of mental ill health.

However, the school staff noted that boys' problems are in some ways more hidden and more comprehensive than one might think. The analysis showed that it was more difficult for the school staff to reach boys when they had suspicions regarding their mental ill health. The boys wanted to engage in discussions with others to a lesser extent and refused to talk to the counselor or the school nurse, which were common desires for girls. Masculinity norms limit boys' opportunities to express themselves, as several of the school staff claimed.

Respondent: It's more shameful in boys, I think. Also perhaps from parents, at home, that they don't dare to tell you that they are unwell. (...) That it might be that as a boy, you shouldn't cry, or something like that. There is still a “macho culture” in society, yes it is more permissive for girls. (School C)

The school staff looked for other signs that could draw their attention to boys who were unwell. They described avoidant or assertive behavior as extremes. On the other hand, some expressed the opposite view and claimed that the differences between boys and girls were not so obvious.

### 4.3 The need to clarify roles and establish a supportive organization

The school staff discussed strategies for dealing with students with mental ill health. This task was complicated when they were uncertain regarding the signs they observed. The analysis provided a clear picture of the unclear division of roles between the school and SHS, conflicting expectations and the lack of available support for the students and school staff. Child and adolescent psychiatry were also mentioned in the discussion of the roles and efforts associated with the various actors.

School staff expressed frustration at the multitude of students who signaled mental ill health. This frustration was related to both time and knowledge. School staff felt they lacked the time necessary to talk properly with the students. Nevertheless, talking to the student in question or the students around him or her was the most common and primary strategy for detecting mental ill health. Talking to the student was also a way to create a bond with the student and determine the next step.

Respondent: But then, I always ask, and then in a calm way. Tell them to stay or something. Hear if there is anything. “I notice you don't ...,” “You seem a little different, has something happened?' Is there something you want to talk about?” “Do you need to talk to someone?” And so on. (School B)

Creating trusting relationships with the students was viewed as an approach that could encourage the students to confide in the teacher if something happened. Staff from SHS claimed that there should be conditions in which adults are present to encourage students to dare to discuss their problems.

Respondent: Trust shapes relationships with adults too, I think. Having good contact with and trust in the adults around you increases the likelihood that you will open up if there is a situation like this, where it is actually not obvious from the outside. But if you don't have adults around you whom you trust, then the probability is less that you will open up about these kinds of issues. (The SHS)

Some members of the SHS highlighted the importance of staff who are easily accessible to students. Only school nurses exhibit “open door accessibility.” School staff (teachers and assistants) can hang around in the school cafeteria or learning studios, thus allowing students to come to them if they need to sit outside their classroom, which served as an example of an accessible environments in which students can discuss their problems. The presence of adults, including clarity regarding who you are, where you are and what you are working with, is crucial for the students' wellbeing, according to this participant. However, more therapeutic conversations should be a responsibility for primary care, as noted by one of the psychologists associated with the SHS.

Lack of knowledge about mental health and ways of addressing mental ill health was a recurring issue mentioned by the school staff. School staff and SHS agreed that school staff lacked sufficient knowledge of how mental ill health should be addressed. However, the SHS claimed that it is primarily insecure teachers who turn to them; nevertheless, the SHS generally held that teachers are talented and have a good grasp of their students' wellbeing.

Overall, the analysis showed that teachers want to be able to meet students and serve as a safe adult; however, they also want to pass the student on to someone within the SHS who has better knowledge. The teachers noted that they did not want to take on the role of SHS. Actors associated with SHS, on the other hand, did not want to take over cases sent by the school staff but rather to provide support for them. The school staff identified their role as that of a fellow human being, not a therapist, because they did not have sufficient knowledge to play the latter role.

Respondent: ...I don't know if there is competence, to be honest. Because it's not... You can feel that yourself, like... yes, we say we talk to our students, but I don't have the skills to do that. I can sit as a fellow human being. I mean, if you came in today, and one of you was sitting there crying, I'd ask “has something happened?” Can I do anything?” That's what I do for the students. I can never go any further than that, because I don't know what it is... It's not my profession. Then you hope that someone else will come along to do it, but they don't. (School C)

Another participant noted that issues pertaining to mental ill health should be addressed by experts and that teachers are not experts. Teachers can be trusted adults to whom students can talk and who can then pass those students on to others who have training in the field.

The analysis highlighted the need for a clearer demarcation for the teaching role as well as the role that the SHS should play. Different views on the role of the SHS were expressed, and the support received from this source was also perceived to vary. The availability of student health personnel varied, and at times, various professions were missing. To address and help students with mental illness, support must be available for both the students and the school staff. Both school staff and the SHS claimed that it takes an excessive amount of time before a student with mental health problems can receive professional help from society. They highlighted the long queue for the regional child and adolescent psychiatry center as a notable problem. The school staff were also unsure about how to treat a student while they were waiting for professional help. They asked for more resources to support child and adolescent psychiatry to ensure that students suffering from mental ill health receive the help they need in time.

Respondent: ... society has few resources connected to this group. If we only think regionally here, it is completely impossible to get into [child and adolescent psychiatry] if you only have a bad mental state. The only reason to come in, it's either if you're directly suicidal or if you've been waiting in line long enough to get a neuropsychiatric evaluation. And it is an extremely big problem that we do not have a society that catches them. (The SHS)

The question of how to provide students with the appropriate form of support was also discussed in relation to child and adolescent psychiatry. School staff expressed skepticism when support for a student with mental ill health was provided by the child and adolescent psychiatry center based solely on second-hand information. They claimed that such advice was given without the professional ever meeting the student.

In the interviews and focus groups, the role of the school was identified as that of providing a space for normality in contrast to a problematic family situation that may lack routines and boundaries. Therefore, the SHS staff also emphasized the importance of students' continuing school during a period of mental ill health, a view that differed from that of the child and adolescent psychiatry, which occasionally calls the student out of school on sick leave.

Respondent: It's really important, I think. We are like one of the biggest protective factors where you have to … So, a functioning school can fill … well, lots of holes. With teachers who see (the student), opportunities to succeed, learn more, have an arena where it is safe and predictable. So... Yes, good school can be survival.

Respondent: At the same time, I also think that there is the fact that school can be the primary cause of mental illness. (The SHS)

Respondents questioned whether it was truly good for the student to be completely away from school since this situation leads to problems when it is time for the student to resume school after a period of no contact. The SHS discussed this situation jointly:

Respondent: Yes, so now come... We have talked about it, the collaboration with [child and adolescent psychiatry]. I think it's sick leave... I won't say lightly. No, but then it's that I don't understand the professions, but that's the way it is. “You must stay at home for at least 2 weeks and have no contact with the school.” Yes, well, I can feel from the school world, we get tied up. “No, she's on sick leave, don't touch,” sort of. (The SHS)

Furthermore, excessive adaptations were questioned because this approach could cause the student to feel singled out.

The results highlight the importance of a well-functioning and stable school organization in which the students' needs for support are met, which in turn also requires support for teachers and student health staff.

## 5 Discussion

This study, which aimed to explore views of staff from schools and school health services on mental ill health among students and their own role in detecting and managing it, resulted in the main theme *Student mental ill health: an increasing problem handled with insufficient resources* as well as the two subthemes *Uncertainty in interpreting students' signs of mental ill health* and *the need to clarify roles and establish a supportive organization*.

The results show that opportunities to address mental health problems were influenced by different types of resources. The school staff's lack of knowledge and confidence with regard to mental health problems and ways of addressing them became evident. Other aspects were a lack of time to talk to students and the problems of collaboration with actors in the health field. The cooperation among the schools, SHS and child and adolescent psychiatry was inadequate, both in terms of the distribution of roles and resources, which led to a difficult and long process that increased the pressure faced by school staff in their attempts to deal with students while waiting for help.

### 5.1 Uncertainty in interpreting students' signs of mental ill health

The school and SHS staff in the present study agreed that mental ill health had increased during recent years, but school staff found it difficult to determine what counted as “real” mental ill health and what were instead signs of other factors in the students' lives. That is, although the school staff observed many signs of mental ill health among the students, they sometimes found it difficult to understand what this term truly meant. This finding is similar to the results reported by Vieira et al. (18) and Shelemy et al. (19), who found that it was difficult for school staff to recognize and identify mental health problems and that they required more knowledge and support to accomplish this goal. The Health Behavior in School-aged Children (HBSC) study (5) indicated an increase in psychosomatic problems since the 1980s, but the results were based on self-reported data. The question of whether the increased reporting of mental health problems among young people reflects a true increase or whether students' experiences of the problems have changed, for example, due to improved awareness of mental health issues in society and an increased tendency to medicalize normal problems, has been debated (24). Some studies have shown that the relevant problems can be bigger than reported by surveys; i.e., adolescents underreport rather than over report mental health problems (25). Another study showed that the measurements of the health of students have exhibited good qualities but naturally failed to account for students who are not at school for some reason (26). Accordingly, there is reason to believe that the reported increase in mental ill health among young people is real, and the increase perceived by the staff in the present study is thereby in line with the increase observed in Sweden in general. This claim is supported by a report from the National Board of Health and Welfare (27) indicating that the number of children and young adults receiving psychiatric outpatient care generally increased during the period 2013–2017. The annual report by Collishaw (28) showed that an increase in mental health symptoms was followed by increased health care consumption. Hermann et al. (29) interviewed adolescents concerning their views of on mental ill health and found that this phenomenon was viewed as increasing and as a normal feature of their lives.

The school staff observed more signs of mental ill health in girls than in boys, a finding which is also in line with previous research that has shown gender differences in, for example, psychosomatic symptoms (5) and anxiety and depression (30). The systematic literature review conducted by Bor et al. (25) found that externalized mental health problems remained stable for adolescent girls and boys but also that girls exhibited an increase in internalized problems. However, no differences were observed in Sweden between boys and girls in regard to the receipt of care for a psychiatric diagnosis or treatment using psychotropic drugs (27). In general, the girls described in the present study were more willing to be open and discuss their problems than were the boys, thus making mental ill health in girls more visible. The boys were described as displaying their mental ill health by, for example, being aggressive or having poor hygiene, but those who merely became silent risked avoiding detection. Research concerning whether, how and why boys and girls express mental ill health in different ways is complex. Previous studies [e.g., (31)] have shown that women suffer from higher rates of depression and anxiety and that men have higher rates of substance abuse and antisocial disorders. However, this finding was not explained by the authors as a result of biological characteristics but rather as the result of social constructs based on different expectations of the genders as well as social structures and power structures related to gender. The school staff in our study did not discuss whether the gender differences they observed were the result of differences in conditions between girls and boys.

According to the results, the school staff questioned some of the students' signs of mental ill health, which can be interpreted as distrust of the students. We argue that the questioning of the students' ill health could be a consequence of the school staff's workload and the difficulty of managing such a large number of students with mental health problems. When signs of mental ill health are framed as socialization using terms such as “contagion” or “sticker” or when students' knowledge of mental health terms is questioned, the problem is reduced; thus, the burden of doing something about this issue becomes less severe. The school staff in the present study were overwhelmed by the prevalence of mental ill health among the students and found this situation to be difficult to manage. This finding can be connected to research that has described the teaching profession as stressful (32), a status which is partly due to the emotional commitment that the work requires (33). A study conducted in Norway showed that the perceived responsibility to help students with mental illness led to feelings of stress, worry and helplessness for female teachers in particular (17). A systematic review (33) and a study conducted in Sweden (34) reported a connection between teachers' emotional commitment and the risk of suffering burnout. This situation may negatively impact the possibilities for teachers to manage students' mental ill health.

However, contagion effects have been found in studies of the associations between mental ill health and social interactions. Based on social contagion theory, some research has highlighted the connections between mental illness and friends, families, and siblings. Some examples of this topic are as follows. Factors that were associated with self-harm among females included exposure to self-harm in friends or family members (35). This claim was also made by a longitudinal study on depression and social networks that was based on data obtained from adult participants in the Framingham Heart Study (FHS) (36). This contagion effect was observed for up to three grades of separation (i.e., the friend of a friend of a friend). Female friends were seen to be particularly influential in the spread of depression from one person to another. The contagion effect has also been found in the context of happiness (37, 38). On the other hand, Eisenberg et al. (39) found only a small contagion association between anxiety and depression only among men in a study of college roommates. These authors also included the contagion effect on happiness in the study but found no evidence to support this claim. Research on the contagion effect has been inconsistent and has thus been criticized (40). Contagion theory risks attributing the problem to the individual, which puts the blame on the individual for his or her own condition. He or she can be blamed for socializing with people who have infected him or her and also bears the responsibility not to infect others; consequently, he or she should avoid social contact. Wester et al. (41) addressed how non-suicidal self-injury (NSSI) resulting from social contagion can be prevented through primary and secondary prevention as well as with tertiary care. Their work was based on, as they noted, the “growing awareness” of contagion with regard to NSSI in schools. Primary prevention includes, for example, emotion regulation, promoting the knowledge of school staff, the provision of clear information to students regarding sources of help and coping methods, as well as a variety of strategies. All strategies refer to the individual level, even if, for example, training is provided to a group or class.

Furthermore, the word “contagion” has been used by adolescents themselves to describe an increased risk of mental ill health due to being surrounded by peers who are unwell (29). This situation was explained as a result of the negative feelings they felt and their perceived responsibility to help. It could be that school staff and adolescents attribute different meanings to the word contagion, such that adolescents view this phenomenon as a normal consequence of being close to friends who are unwell, while school staff perhaps view it as a sign that not all expressions of mental ill health must be taken seriously.

A study of the terms and concepts used for mental health among adolescents showed that 15-year-olds used certain terms for psychiatric diagnoses, such as anxiety and depression, to describe everyday issues and strains, such as their worries about failing a test or situations in which they felt somewhat low (42). On the other hand, young people themselves make distinctions between anxiety and real anxiety; the same is true of depression (42). Lindholm and Wickström (42) further noted that adolescents use these terms as cultural categories in a manner that was detached from pathology; instead, they used them as a way of addressing relevant issues in their lives. In the present study, the SHS asked for common definitions of mental health terms used by students, the participants themselves, and other school staff. According to Lindholm and Wickström (42), another way to understand this situation could be that school and SHS staff must be more aware of how students use mental health terms as cultural categories.

### 5.2 The need to clarify roles and establish a supportive organization

The school staff in the present study believed that it was important to establish good relationships with the students to help them communicate about mental ill health should problems arise. This claim is in line with a study conducted by Nesset Mælan et al. (43) who found that the lower secondary school teachers in their study viewed themselves as “dialog partners” to the students, which entailed talking about subjects other than schoolwork. This approach was viewed as a way to support students' mental health. A study that included students and supporting school staff as well as teaching staff also highlighted the importance of trusting relationships to encourage students to turn to the staff with their issues (44). An example from the current evidence base on approaches to building trusting teacher-student relationships shows that understanding one's students is an important part of relationship building and that teachers' ability to take their students' perspectives led to improved relationships between teacher and student (45).

The staff in the study conducted by Nesset Mælan et al. (43) further described a tension between their teaching role and obligations and their ability to take the time to talk to the students, a dilemma that is also described by the school staff in the present study in situations in which time was a limited resource. Working with young students involves a balancing act between the role of teacher and that of a fellow human being, which can sometimes be difficult. Another dilemma faced by school staff is the boundaries between the teaching role and that of a therapist are sometimes unclear (43). The school staff in both Nesset Mælan et al. (43) and the present study discussed issues relating to those unclear boundaries. Shelemy et al. (19) found that teachers did not want to take on the role of therapist. Although school staff must consider students' needs with regard to mental health, they are not mental health professionals. Several of the staff in the present study noted that they did not want to perform work that, according to them, should instead be performed by the SHS.

The results of the present study showed that the school staff took on responsibilities related to students' mental ill health for which they claimed that they were not educated and that were not primarily in the scope of their role as teachers. SHS staff expressed concern that teachers feel they are working outside their area of expertise, and they must thus be comfortable and confident in promoting and teaching in support of mental health. Gaps in teacher training and continuing professional development in this respect have been a notable challenge identified in the literature (46). The extra burden faced by school staff with regard to the need to address (suspicions of) mental ill health among students represents a risk in their working environment (17), and a lack of support for school staff on the part of health professionals with regard to managing student mental ill health ultimately affects students' mental health. The lack of capacity and the absence of communication with mental health professionals has been described in previous research. For example, participants in the study conducted by Shelemy et al. (19) asked for better communication with child and adolescent mental health services, and the studies conducted by Sharpe et al. (47) and Mansfield et al. (48) identified a lack of mental health service capacity as a barrier to helping students with mental ill health in schools in England.

An explanation for the role conflicts and cooperation problems between the school and SHS in the present study may be that the role of SHS has changed from having had a main task of working with prevention, vaccinations, screening and focus on children's development to a more holistic task (21). At the same time, psychological problems for young people have increased (3, 5, 6). In many countries, the focus is still on screening and prevention (21). In Sweden, SHS' mission since 2010 has been to promote health, according to the School Act (20). This means an increased demand for cooperation for student health, and a responsibility for all school staff to promote student learning and health. In Sweden, for many years, the Swedish National Agency for Education has worked to make student health a common mission for the school, but the results of our study show that there is still much to be done. The increasing mental ill health among students places great demands on the school and SHS, while in Sweden, similar to for example England (49), it has become increasingly difficult for children and young people to get help from psychiatry. Both the SHS and the school staff address the problem of long waiting times in child and youth psychiatry and that a student almost needs to be suicidal to receive care. This means that the school's mission to promote health is limited when the resources have to be used to deal with the students' mental ill health.

In summary, the school staff discussed this tangle of mental health problems and ways of addressing this situation from a more reductionistic standpoint rather than from a system perspective. The contagion effect was viewed as a part of this issue. From this perspective, the risk of blaming parents, students and even colleagues increases. Members of the school staff face the pressure of addressing all students who exhibit signs of mental ill health, and these problems are currently spreading like a pandemic. However, the results also show that the school staff wanted the school to represent a safe and continuous everyday life and to promote normality; in addition, teachers did not want to take on the role of therapists but rather to be able to contribute to good interpersonal relationships. They wanted their students to be able to access professional support from specialists.

Members of the school staff wanted a school that provides support in everyday life. They discussed the idea that the school can be a counterbalance to a chaotic home environment with the aim of promoting good social relations and opportunities to learn. This claim is in line with St. Leger's (1) discussion of recent developments in HPS. Namely, HPS has shifted from a desire on the part of the health sector to view the school as an arena for health promotion to the school's view of student health and wellbeing as a prerequisite for learning. In our study, instead of being able to provide an environment that can promote health and learning, the teachers struggled with the signs of mental ill health and ways of addressing this situation as well as the frustration that occurred when collaboration with health professionals was unsuccessful.

The building blocks for HPS (50), which were developed years ago and involve schools' development of a health policy, establishment of a good physical and social environment, and creation of personal health competence and a health plan, are all important factors for the pursuit of healthier schools. Based on the present study, we want to highlight two aspects of this process: cooperation with the local community, integrated health care and a supportive infrastructure. Mental health and wellbeing among young people are the responsibility of the local community, and the work must be performed in collaboration. Our results highlight the fact that the school's staff feel alone when engaging in this work. The infrastructure is insufficiently supportive for either the school staff or the students, and the SHS must be more integrated than it currently is. For example, the time between finding out that a student is in need of professional help and the time when the student receives such help is currently unreasonably long.

St. Leger (1) addressed the fact that schools that address students' social and emotional wellbeing can more easily see the connection between health and learning. Therefore, it is important for the health sector to strengthen schools' ability to promote students' health by providing them with evidence regarding how to improve both health and educational outcomes. Finally, St. Leger highlighted the importance of providing ongoing professional development for teachers.

### 5.3 Methodological discussion

The qualitative design of this study, which featured focus groups, individual interviews and a thematic analysis in line with Braun and Clarke (23), was suitable for its goal to explore views of staff from schools and school health services on mental ill health among students and their own role in detecting and managing it. The strengths of the study include the fact that two researchers were involved in both data collection and analysis, which facilitated a continuous process of developing and refining the results of the analysis through dialog. Competing interpretations were discussed. According to Graneheim and Lundman (51), this approach can increase the credibility of the findings. To assess resonance (52), the findings concerning the contagion effects of mental ill health at school were discussed with practitioners and researchers at conferences during the analysis, who confirmed these findings. Furthermore, to the best of our ability, we must be clear about whose voice is expressed, i.e., that of the participants or our own interpretations as researchers, an approach which increases the study's trustworthiness according to Graneheim et al. (53). Selected quotes were included in the presentation of the results for this purpose.

The present study presents a secondary analysis of data that was initially collected on two occasions with the aim of evaluating an intervention; however, somewhat different interview guides were used on the first and second occasions. This inconsistency could affect the dependability of the study (51). More general questions that did not pertain to the intervention were asked initially during the focus groups and interviews, for example, questions regarding the participants' views of trends and signs of mental ill health among the students. Since extensive discussions concerning these issues took place and we found the findings interesting, we decided to analyze the material in line with the purpose of this study. The possibility that the intervention affected the results cannot be ruled out; however, we did not perceive that the participants discussed these issues differently before and after the intervention.

According to Malterud et al. (54), the number of participants required in qualitative research can be determined based on the concept of information power, which is, among other factors, dependent on the breadth of the aim, the quality of the dialog, the analytical approach and the application of an established theory (or lack thereof). We perceived the dialog in this study to be of high quality and observed that the 40 participants were a sufficiently large sample considering the aims of this research and the inductive analytical approach. If this study had featured a comparison between before and after the intervention, the fact that the two rounds of data collection did not include the same participants could be viewed as a limitation. Instead, the participants at each round were viewed as representing the experiences of school staff at that time. Considering the aim of this study, this fact could rather represent the strength of this research, as it allowed us to collect the perspectives of more individual participants. The amount of data thus collected offered us a thick description and allowed saturation to be achieved, which became evident in round two of the data collection process. However, the possibility that staff who are chose to not participate had different views about student mental health cannot be ruled out.

Another aspect that may have affected the dependability and credibility of this study is that data were collected in different ways on the two occasions. In the first round, onsite focus groups were conducted at the three schools and the SHS. Due to the small number of participants in some of the focus groups, staff members who could not participate because they were busy at that time were asked to participate in individual interviews at a time of their choosing. Four people chose to participate individually either online or by telephone. In the second round of data collection, the restrictions resulting from the pandemic hindered onsite meetings, and all data were collected via a video conference platform. No individual interviews were conducted on this occasion. The different means used to collect the data entailed that the participants participated in the research under different conditions; however, our view is that this factor did not affect the data to any large extent. The pandemic led to an increased use of digital solutions, and most staff were accustomed to digital tools at the time of round two.

It is up to the reader to decide whether the results of this study are transferable to their own context (51). The fact that this study was conducted in a rural municipality in Sweden, which features large geographical distances among the schools, the students' homes and the child and adolescent psychiatry facility, should be noted. This factor could have affected how the school staff and SHS viewed the possibility of collaboration with other health actors and the opportunities for the students to receive help.

## 6 Conclusions

To conclude, opportunities for school staff to address mental health problems among students are influenced by different aspects of resources. School staff are often uncertain regarding how to interpret signs of mental ill health, and in this study, they found the number of students with mental ill health to be overwhelming. It is necessary to promote better knowledge among the school staff about mechanisms related to mental ill health as they were unsure of what counted as “real” mental ill health and what were instead signs of other factors in the students' lives. Furthermore, schools must be organized to manage students' mental ill health more effectively without placing responsibility on teachers, who need better conditions to fulfill their core mission. Accordingly, necessary factors in this context include more available health professionals as well as a consensus regarding support for students with mental ill health in terms of the division of responsibilities between school staff and the SHS. The results showed that the cooperation between the schools, SHS and child and adolescent psychiatry was inadequate, both in terms of the distribution of roles and in terms of resources, which led to a difficult and long process for the student and also increased the pressure faced by the teachers with regard to how to treat the student while waiting for help.

The reductionistic and problem-based mindset of the school is still the dominant perspective, although the primary tasks of SHS pertain to prevention and health promotion. Changing this perspective by adopting a whole-system approach with a focus on health promotion can help schools address the mental health of students in a more supportive way.

## Data availability statement

The datasets presented in this article are not readily available because of ethical reasons. Requests to access the datasets should be directed to ÅS, asa.m.svensson@miun.se.

## Ethics statement

Ethical approval was not required for the studies involving humans because the participants were interviewed in their profession. The studies were conducted in accordance with the local legislation and institutional requirements. The participants provided their written informed consent to participate in this study.

## Author contributions

ÅS: Conceptualization, Formal analysis, Investigation, Methodology, Writing – original draft, Writing – review & editing. MW: Conceptualization, Formal analysis, Investigation, Methodology, Writing – original draft, Writing – review & editing.
